# Impact of the COVID-19 pandemic on global health research training and education

**DOI:** 10.7189/jogh.10.020366

**Published:** 2020-12

**Authors:** Lifang Hou, Supriya D Mehta, Elizabeth Christian, Brian Joyce, Olufunmilayo Lesi, Rose Anorlu, Alani Sulaimon Akanmu, Godwin Imade, Edith Okeke, Jonah Musah, Firas Wehbe, Jian-Jun Wei, Demirkan Gursel, Kate Klein, Chad J Achenbach, Ashti Doobay-Persaud, Jane Holl, Mamoudou Maiga, Cheick Traore, Atiene Sagay, Folasade Ogunsola, Robert Murphy

**Affiliations:** 1 Department of Preventive Medicine, Division of Cancer Epidemiology and Prevention, Feinberg School of Medicine, Northwestern University, Chicago, Illinois, USA; 2 Institute for Global Health, Feinberg School of Medicine, Northwestern University, Chicago, Illinois, USA; 3 Division of Epidemiology and Biostatistics, School of Public Health, University of Illinois at Chicago, Chicago, Illinois, USA; 4 Department of Medicine, College of Medicine of the University of Lagos, Lagos, Nigeria; 5 Department of Obstetrics and Gynecology, College of Medicine, University of Lagos, Lagos, Nigeria; 6 Department of Haematology and Blood Transfusion, Lagos University Teaching Hospital, and College of Medicine of the University of Lagos, Lagos, Nigeria; 7 Department of Obstetrics and Gynecology, College of Health Sciences, University of Jos, Jos, Nigeria; 8 Department of Preventive Medicine, Division of Health and Biomedical Informatics, Feinberg School of Medicine, Northwestern University, Chicago, Illinois, USA; 9 Department of Pathology, Feinberg School of Medicine, Northwestern University, Chicago, Illinois, USA; 10 Division of Infectious Diseases, Department of Medicine, Feinberg School of Medicine, Northwestern University, Chicago, Illinois, USA; 11 Department of Medicine, Feinberg School of Medicine, Northwestern University, Chicago, Illinois, USA; 12 Biological Sciences Division, University of Chicago, Chicago, Illinois, USA; 13 School of Professional Studies, Northwestern University, Chicago, Illinois, USA; 14 University of Sciences, Techniques and Technologies of Bamako (USTTB), Bamako, Mali; 15 Department of Medical Microbiology, College of Medicine, University of Lagos, Lagos, Nigeria

Since January 2020 billions of people across the world have been “locked down” and hundreds of thousands have died because of the COVID-19 global pandemic. The pandemic is creating enormous adverse economic and social consequences throughout the world, with direct and indirect impacts on global health activities - particularly through the participation of health care workers, clinicians, investigators, technologists, and students in research training and educational programs in low- and middle-income countries (LMICs).

A primary approach of global health training programs has been to bring trainees to grantee institutions in developed countries to participate in in-person coursework, attend skills-based workshops, and/or work directly with researchers and mentors. Another important component has been travel by mentors in the grantee institutions to partner institutions in LMICs to facilitate workshops, lectures, and in-person clinical investigation and laboratory trainings. Training has also consisted of participation in formal courses and degrees, with trainees completing either full-degree or certificate programs at universities, and/or working directly with their mentor. However, the near-complete shutdown of international travel prevents faculty, mentees, and mentors from taking part in these indispensable exchanges.

## CURRENT AND FUTURE CHALLENGES

One of the largest institutions funding this type of education and training is the United States (US) National Institutes of Health (NIH) Fogarty International Center (FIC), dedicated to supporting and facilitating partnerships between health research institutions in the US and LMICs around the globe, and training scientists to address global health needs [1]. FIC has funded 153 global health training programs, including 118 programs that were active as of April 2020 [2]. FIC D43 training programs are funded through peer-reviewed grants and designed to be collaborative, long-term, and flexible to meet the research priorities of both the US and foreign institutions [2]. The training goals of a D43 program include short-, medium- and long-term goals. Short-term training goals are designed to be accomplished in less than three months and are usually composed of faculty-led workshops or training sessions focused on specific areas of research methods or laboratory skills [3]. Medium-term training goals are designed to be accomplished over three to six months and include components such as working directly with a faculty mentor on a research project and taking non-degree courses to support specific research topics [3]. Long-term training goals are designed to be accomplished over a period of six months or more and can include formal graduate education such as master’s, doctoral, and post-doctoral degree programs related to the training areas needed and public health concerns within trainees’ home countries [3].

**Table 1** provides information on strengths and weaknesses reflecting the impact that the COVID-19 pandemic may have on activities of global training programs based on our own experiences learned from our NIH FIC D43 training program in Nigeria and other global health educational programs. Short-term training activities previously had in-country, on-site components requiring travel by US faculty and mentors, which resulted in significant team-building and socio-cultural adoption. Due to COVID-19, travel is postponed or cancelled, replaced by shorter and more frequent virtual communications and lectures. This paradigm shift has resulted in lower program expenditures, more frequent contact between trainers and trainees, and allowed for the recruitment of a wider range of faculty for educational activities than would normally be feasible. Conversely, online interactions represent a loss of opportunity to expose mentees to other cultures and a diversity of ideas only available through international travel, including learning about different approaches to work and study methods, making new friends, starting networks potentially outside their original trainee study groups, and building global citizenship.

**Table 1:** Challenges and opportunities of global health training program due to COVID 19 pandemic*

Pre-COVID-19			During COVID-19		
**Planned Activities**	**Weaknesses**	**Strengths**	**Adapted Activities**	**Weaknesses**	**Strengths**
**Short-term training programs (0-3 months-duration): conferences/symposia, workshops, faculty enrichment; 1-3 participants in the US, 1-30 in-country**					
On-site workshop training	Requirement of travel	Direct in-person contact	Replaced by video presentations and discussions led by US faculty	Lack person-to-person interactions	Expenses reduced
	Costly				Able to spread out for more contact over longer time
	Limited frequencies	Cross-institution team building		Lack of experience in US institute	Can source lectures from more faculty without cost barrier of travel
	Social-cultural adoption				
On-site annual meeting	Requirement of travel	Direct In-person contact	Replaced by video meetings	Lack person-to-person interactions	Expenses reduced
	Costly				
	Limited frequencies			Lack of experience in conferences and meetings	
	Social-cultural adoption	Cross-institution team building			More US and LMIC faculty are able to join without cost barrier of travel
Faculty Enrichment	Requirement of travel	Cements cross-institution collaboration	Postponed	Rely on email and other online communications	None
	Costly				
**Medium term: specialized training (3-6 months)**					
Specialized training	Requirement of travel	Able to focus on specific research skills for competencyD	50% of trainees postponed	Loss of direct contact with faculty	Expenses reduced
					Trainees joined virtual lectures for increased contact with US faculty
	Costly	irect In-person contact	50% of trainees were able to participate in online nondegree training		More trainees able to join virtual sessions
**Long Term: Graduate degree and non-degree training (>6 months)**					
Masters (Non home in-person)	Social-cultural adoption	Advanced research training in variety of skills	Trainees that already started continued	Loss of direct contact with faculty	Expenses reduced for those transitions online Trainees joined virtual lectures for increased contact with US faculty
	Must set up LMIC in-country research from US		Trainees expected to start were transitioned to online per university	Loss of interaction with other students	
Masters (US online)	Less direct contact with US faculty	Advanced research training in variety of skills	Trainees continued as university adjusted	None	Trainees joined virtual lectures for increased contact with US faculty
		Access to NU online resource			
PhD (US in-person)	Must set up LMIC in-country research from US	Social-cultural learning	Trainees that already started continued	Loss of direct contact with faculty	Expenses reduced for those transitions online
		Advanced research training in variety of skills			
		Access to NU resources	Trainees expected to start were transitioned to online per university	Loss of interaction with other students	Trainees joined virtual lectures for increased contact with US faculty

LIMC – low- and middle-income countries

*Based on Northwestern University Fogarty International Center (FIC) funded D43 global health training programs (full list of training grants can be found at https://www.globalhealth.northwestern.edu/centers/communicable-diseases/index.html and https://www.globalhealth.northwestern.edu/centers/oncology/index.html).

**Figure:**
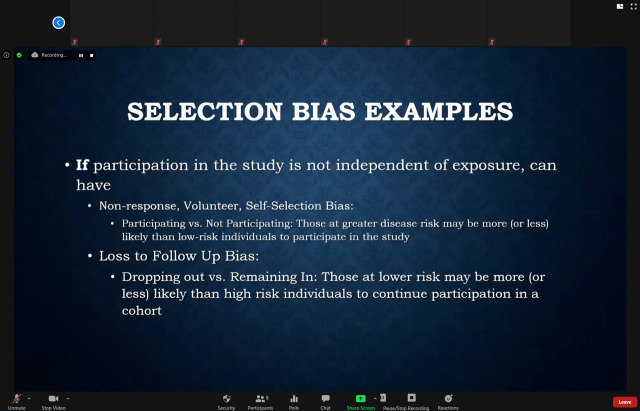
Photo: From the authors’ own collection, used with permission.

Medium-term training activities were often accomplished through a mix of in-person training in the US and LMICs. Trainees take part in specialized training with US-based faculty, with either party traveling, followed by in-country research. These medium-term training activities have similar weaknesses and strengths to the short-term training activities in response to the COVID-19 pandemic. For longer-term training activities, the COVID-19 pandemic is not projected to substantially impact in-country research and graduate training beyond the loss of travel for US and in-country partners and the delay in trainings that are absolutely needed in person. Many global health research training programs were already shifting online and can continue uninterrupted; traditional US-based graduate training is quickly catching up, with most already offering some online curriculum materials to trainees who are unable to travel to the US. While the COVID-19 pandemic may have a lasting impact on global health training and education, at the same time, these challenges can provide new opportunities.

## OPPORTUNITIES IN A POST-COVID WORLD

The COVID-19 pandemic has forced global health research education and training program leaders and educators to consider and explore opportunities available through distance learning. Below, we give some examples from our programs that could hint at future transformations.

### Virtual education lectures

Our own experience using a technological solution to replace traditional, in-person lectures has been well-received. A pilot presentation was set up for a US-based faculty member to give an introductory lecture on one of our project core competencies, cancer epidemiology. The lecture information and instructions to access and use the web-based video teleconferencing system were shared with two Nigerian sub-sites. In real time, attendees listened to the lecture, viewed the slides, and interacted with and provided feedback to the lecturer, facilitated by including defined opportunities for questions and discussion. To fully participate in the discussions, attendees had to ensure they had a secure internet connection. Conferencing etiquette skills such as muting of participants to preserve audio quality, use of hand-raising functions, and control of video view initially stifled open discussion but were rapidly adopted. Attendees expressed their enthusiasm for being able to attend in real time and having the opportunity to interact with the instructor. The lecture series has continued successfully as monthly sessions. Using remote lectures allowed mentors to connect with trainees at partner sites and accommodate more trainees, and enables lectures from more professors.

### Telemedicine approaches

Traditionally, pathology training has been conducted under a microscope in a trainee’s home institution. Under COVID-19 travel restrictions, we have been using digital or telepathology technologies to provide training sessions to our pathologist trainees. Pathological slides are translated into digital or virtual images using an image scanner and shared by mentors and mentees from a desktop/laptop computer without restrictions to geographic location with no loss of information. The image can be stored in a cloud and accessed and reviewed any time by both mentors and mentees, at the same time, with no additional laboratory equipment. Similar approaches can be used in other medical training programs, such as digital radiology and other telemedicine.

### Molecular laboratory training and education

The training of molecular laboratory scientists has largely been conducted in local laboratories, or by bringing trainees to the host institution(s). Since the COVID-19 outbreak, we have used virtual and digital technologies to train molecular laboratory trainees at our partner universities in Nigeria in both theoretical and practical perspectives with excellent feedback and promising results. These virtual approaches enable us to streamline the complicated tasks common to molecular laboratories such as development and implementation of protocols and quality control (QC) procedures, sample collection and processing, inventory and tracking, data entry, and load balancing. Such virtual cross-talk allows mentors and mentees to connect via video, and allows the mentees to see each protocol step in detail regardless of geographic proximity.

### Training health care providers

Before the COVID-19 pandemic, Northwestern University and the University of Lagos in Nigeria had been planning a user-centered palliative care training program for health care professionals without formal training in Nigeria. We initially planned a 2-day on-site workshop leveraging the previously implemented content from the EPEC (Education in Palliative and End-of- Life Care) program[4] which trains physicians, nurses, and other health care professionals in fundamental palliative care skills in communication, ethical decision-making, psychosocial considerations, and symptom management. Because of the COVID-19 pandemic, we have redesigned the curriculum and workshops. Lecture, video, role plays, and small group discussions will be held virtually. To enhance the interactions between trainees and mentors, we use breakout room features for small group discussion and chat functions to support interactive question and answer periods. Other online apps or interactive software for polling, whiteboard, and extension technology functions are also adopted to enhance the interactions.

As shown by these examples, the COVID-19 pandemic has transformed global health training and educational programs. While virtual approaches can be effective at lower cost, they may also have deleterious effects on trainees’ mentorship and professional development as a result of weakened or lost in-person interactions, team building, socio-cultural adoption and understanding, and interpersonal relationships [5]. Given the altered formats and timelines of training as demonstrated in **Table 1** and the possibility of long-term adoption of these changes, future programs may attract different types of learners with different and varied motivations, expectations, and outcomes [6]. The development of new “hybrid” models using proven virtual components but with built-in, in-person activities better suited to discussion, idea generation, and team and relationship building may provide the best approaches for global health training and education in the post COVID-19 era.
